# Relationship between obesity and unrecognized myocardial infarction: a EuroCMR multi-center study

**DOI:** 10.1186/1532-429X-15-S1-O76

**Published:** 2013-01-30

**Authors:** Christoph J Jensen, Brenda Hayes, Michele Parker, Anja Wagner, Massimo Lombardi, Juerg Schwitter, Oliver Bruder, Heiko Mahrholdt, Raymond J Kim

**Affiliations:** 1 Duke Cardiovascular Magnetic Resonance Center, Duke University, Durham, NC, USA; 2 Comprehensive Cardiology of Stamford and Greenwich, Stamford, CT, USA; 3 Cardiovascular MR Unit, C.N.R. Regione Toscana G. Monasterio Foundation, Pisa, Italy; 4 Department of Cardiology, University Hospital Lausanne, Lausanne, Switzerland; 5 Department of Cardiology and Angiology, Elisabeth Hospital, Essen, Germany; 6 Department of Cardiology, Robert Bosch Medical Center, Stuttgart, Germany

## Background

Obesity is a major public health issue given its high cardiovascular morbidity and mortality. However, whether obesity predicts cardiovascular disease independent from traditional Framingham risk factors is controversial [1]. Delayed-enhancement CMR (DE-CMR) allows for sensitive and specific detection of unrecognized MI, which appears associated with adverse prognosis similar to clinically recognized MI.

We examined the relationship between obesity and the prevalence of unrecognized myocardial scar (UScar) and/or unrecognized MI (UMI) as determined by DE-CMR.

## Methods

This is a study of the EuroCMR registry. Patients with suspected CAD (but without documented CAD) undergoing CMR at 17 centers (in 6 countries) were enrolled. Between April-2009 to April-2012, clinical and CMR data were submitted to the EuroCMR Data Coordinating Center in 1508 consecutive patients. Incomplete datasets (N=78) and patients with documented vascular disease (N=93) were excluded. All CMR findings were validated at an imaging corelab blinded to patient identity and clinical information. Scar was defined as any hyper-enhancement, MI as CAD-pattern hyperenhancement on DE-CMR. For each patient, Framingham risk score (FRS) for prediction of 10-year general cardiovascular disease was calculated.

## Results

**Figure 1:**
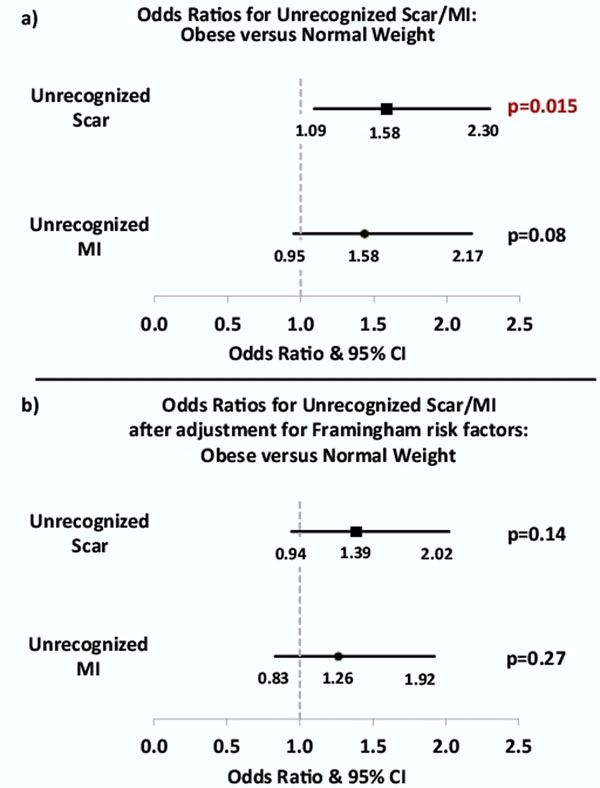
Odds ratios for unrecognized scar and unrecognized MI for obese (BMI ≥25 kg/m^2^) versus normal weight (BMI <25kg/m^2^). Part A shows the unadjusted odds ratios, whereas part B shows the Odds ratios for unrecognized Scar and unrecognized MI after adjustment for Framingham Risk Score not including weight.

**Figure 2:**
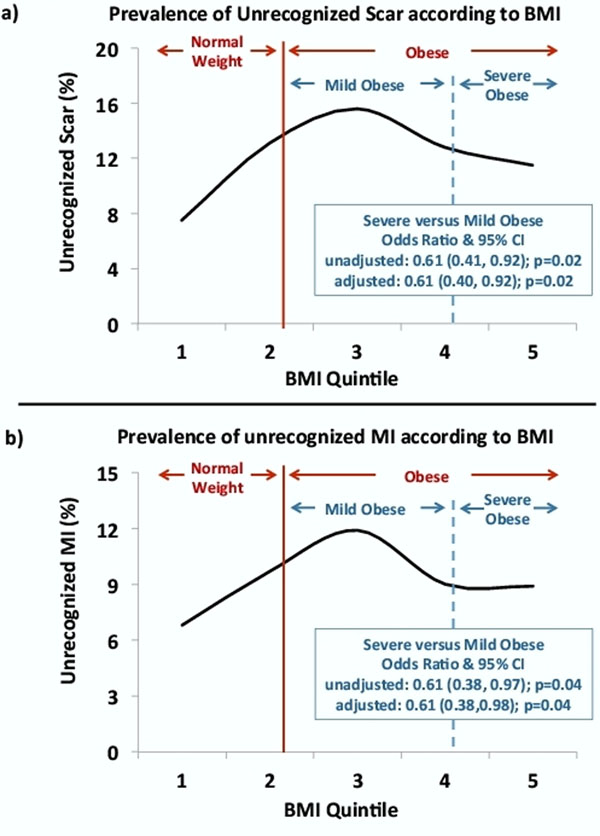
Prevalence of unrecognized scar (a) and unrecognized MI (b) in relation to BMI groups. BMI groups are expressed as quintiles of the study population. The red line separates patients with normal weight (BMI <25 kg/m^2^) from patients with obese (BMI ≥25 kg/m^2^). The dashed blue line depicts the cut-off (29.5 kg/m^2^), which separates the mildly obese from the severe obese patients. Odds ratios for UScar and UMI for severe obese versus mild obese are shown, unadjusted and adjusted for Framingham Risk Score not including weight.

1337 patients (60±13 years; 59% male), 461 normal weight (BMI<25 kg/m^2^) and 876 obese (BMI≥25kg/m^2^), were included. The overall prevalence of UScar and UMI was 12% and 9%, respectively. Obese patients had higher prevalence of UScar compared to normal weight patients (13.7% vs 9.1%, p=0.015), and a similar trend was observed for UMI (10.3% vs 7.4%, p=0.08, Figure 1a). Prevalence of UScar and UMI increased substantially with increasing FRS (both P_(trend)_<0.0001; first quintile FRS: UScar/UMI= 4.9%/3.0%; fifth quintile FRS: UScar/UMI= 18.4%/13.9%). After adjustment for FRS, the higher prevalence of UScar in obese patients was no longer significant (Figure 1b). The prevalence of UScar and UMI in relation to weight showed a convex-curve (Figure 2), peaking in the mildly obese and dropping for more significantly obese. Traditionally, BMI ≥30kg/m^2^ defines true obesity from merely overweight (i.e. severe vs mild obesity). Using a cutpoint of 29.5 kg/m^2^, those with severe obesity were less likely to have UScar and UMI than those with mild obesity (both p<0.05, Figure 2). This difference persisted after adjusting for FRS.

## Conclusions

Overall, obese patients are not more likely to have unrecognized scar or MI than normal weight patients after adjustment for Framingham risk factors. However, among obese patients, those more severely obese have *reduced* rates of UScar and MI compared with those less obese, which suggests an obesity paradox.

## Funding

None.
